# Integrated Physiological, Transcriptomic, and Metabolomic Analysis Reveals Mechanism Underlying the *Serendipita indica*-Enhanced Drought Tolerance in Tea Plants

**DOI:** 10.3390/plants14070989

**Published:** 2025-03-21

**Authors:** Gaojian Shen, Hongli Cao, Qin Zeng, Xiaoyu Guo, Huixin Shao, Huiyi Wang, Liyong Luo, Chuan Yue, Liang Zeng

**Affiliations:** 1 Integrative Science Center of Germplasm Creation in Western China (CHONGQING) Science City, College of Food Science, Southwest University, Chongqing 400715, China; 2 Chongqing Key Laboratory of Speciality Food Co-Built by Sichuan and Chongqing, Southwest University, Chongqing 400715, China

**Keywords:** *Camellia sinensis*, *Serendipita indica*, drought stress, transcriptome, metabolome

## Abstract

Drought stress significantly impairs the output of tea plants and the quality of tea products. Although *Serendipita indica* has demonstrated the ability to enhance drought tolerance in host plants, its impact on tea plants (*Camellia sinensis*) experiencing drought stress is unknown. This study assessed the response of tea plants by inoculating *S. indica* under drought conditions. Phenotypic and physiological analyses demonstrated that *S. indica* mitigated drought damage in tea plants by regulating osmotic equilibrium and antioxidant enzyme activity. Metabolome analysis showed that *S. indica* promoted the accumulation of flavonoid metabolites, including naringin, (-)-epiafzelechin, naringenin chalcone, and dihydromyricetin, while inhibiting the content of amino acids and derivatives, such as homoarginine, L-arginine, N6-acetyl-L-lysine, and N-palmitoylglycine, during water deficit. The expression patterns of *S. indica*-stimulated genes were investigated using transcriptome analysis. *S. indica*-induced drought-responsive genes involved in osmotic regulation, antioxidant protection, transcription factors, and signaling were identified and recognized as possibly significant in *S. indica*-mediated drought tolerance in tea plants. Particularly, the flavonoid biosynthesis pathway was identified from the metabolomic and transcriptomic analysis using Kyoto Encyclopedia of Genes and Genomes (KEGG) enrichment analysis. Moreover, flavonoid biosynthesis-related genes were identified. *S. indica*-inoculation significantly upregulated the expression of *cinnamate 4-hydroxylase* (*C4H*), *chalcone synthase* (*CHS*), *flavanone 3-hydroxylase* (*F3H*), *dihydroflavonol 4-reductase* (*DFR*), *anthocyanidin reductase* (*ANR*), and *leucoanthocyanidin reductase* (*LAR*) genes compared to uninoculated plants subjected to water stress. Consequently, we concluded that *S. indica* inoculation primarily alleviates drought stress in tea plants by modulating the flavonoid biosynthesis pathway. These results will provide insights into the mechanisms of *S. indica*-enhanced drought tolerance in tea plants and establish a solid foundation for its application as a microbial agent in the management of drought in tea plants cultivation.

## 1. Introduction

Drought stress is one of the significant challenges for global food security and agricultural productivity, directly limiting crop growth and reducing yields [1]. The anticipated effects of climate change are likely to intensify the frequency and extremity of drought events. Fortunately, plants have evolved adaptive mechanisms involving morphological, physiological, genetic, and metabolic modifications to cope with drought stress [2]. Drought activates osmotic substances and antioxidant enzymes in plants, including soluble proteins, soluble sugars, proline, superoxide dismutase (SOD), catalase (CAT), and peroxidase (POD), to improve survival capacity [3]. Flavonoids, ubiquitous secondary metabolites in plants, are essential for responding to adverse environments. They function through mechanisms such as reactive oxygen species (ROS) scavenging, signal transduction, regulation of gene expression, metabolic reprogramming, stomatal movements, and photosynthesis [4]. It has been well documented that the expression of flavonoid biosynthesis-related genes is upregulated, and flavonoids accumulate in response to drought stress [5,6,7].

Currently, to mitigate drought stress, various strategies have been implemented, such as breeding drought-resistant varieties, optimizing cultivation techniques, and applying exogenous substances [8]. There is increasing interest in adopting new approaches to green agriculture and sustainable development, particularly by leveraging the natural interactions between plants and microorganisms. Microorganisms can stimulate the growth potential of plants, enhance drought and disease tolerance, and remediate heavy metal pollution, showing great application potential and value in agriculture [9]. Kumar et al. [10] demonstrated that the combination of *Pseudomonas* sp. TR15a and *Bacillus aerophilus* TR15c promoted the growth of sunflower in copper-contaminated soil by producing indole acetic acid (IAA), siderophores, and dissolving phosphates. *Bacillus licheniformis* K11 enhanced drought stress tolerance in pepper plants by upregulating the *expression of dehydrin-like protein* (*dhn*), *plant small heat shock protein* (*sHSP*), *vacuolar H^+^-ATPase* (*VA*), and *pathogenesis-related protein 10* (*PR-10*) genes [11]. Additionally, arbuscular mycorrhizal fungi (AMF) *Funneliformis mosseae* mitigated the drastic downregulation of photosynthesis-related genes and the reduction of photosynthetic CO_2_ assimilation rates caused by cucumber mosaic virus (CMV) infection, thereby influencing the development of CMV infection in tomato plants [12].

*S. indica*, a root endophytic fungus of the order Sebacinales (Basidiomycota) [13], was first isolated from xerophytic plant roots in the Thar Desert of India and is characterized by its pear-shaped chlamydospores [14]. Its spores are commonly observed within the epidermal and cortical cells of host plant roots [15]. *S. indica* can be cultivated in vitro on synthetic media. It promotes plant growth, enhances crop yield, improves fruit quality, and boosts resilience to biotic and abiotic stresses by inducing systemic resistance, augmenting antioxidants, optimizing nutrient use, and modulating plant hormones [16,17]. Recently, studies on wheat [18], rice [19], maize [20], cucumber [21], and tomato [22] plants have demonstrated that inoculation with *S. indica* increased SOD activity, proline content, chlorophyll levels, and water use efficiency, regulated stomata behavior, and diminished malondialdehyde (MDA) levels under water-deficient conditions, thereby improving the drought tolerance of host plants. The enhanced drought tolerance conferred by *S. indica* is mainly attributed to the increase in hormone and proline contents, effective ROS scavenging, and the induction of the expression of drought-responsive genes [23]. For example, *S. indica* decreased ROS levels and increased POD and CAT activities in wheat leaves under drought stress [24]. In drought-exposed cabbage, *S. indica* inoculation boosted the expression of drought-associated genes, namely, *DREB2A*, *CBL1*, *ANAC072*, and *RD29A* [25]. However, the understanding of the impacts of *S. indica* on the drought stress response in woody species, including tea plants, is limited.

Tea plants, an economically vital crop in tropical and subtropical regions, contain unique metabolites that endow them with rich flavor and health benefits and are typically cultivated in rain-fed agricultural systems [26]. It has been reported that drought stress reduced tea yield and elevated the mortality rate of tea plants [27]. Recently, diverse omics technologies, including transcriptomics, metabolomics, and proteomics, have been extensively employed to decipher drought adaptation mechanisms in tea plants. These studies unveil that the tea plant response to drought is multifaceted and complex, involving transcription, transcript processing, translation, post-translational modifications, and epigenetic modifications [28,29]. Interestingly, several studies have demonstrated that microorganisms play an important role in protecting tea plants against drought stress [30,31]. *S. indica*, as a beneficial fungus, demonstrates substantial potential for improving drought tolerance in agricultural systems [32,33]. Rong et al. [34] reported that *S. indica* is capable of colonizing tea plants and promoting phosphorus acquisition under phosphorus-deficient conditions. Cernava et al. [35] discovered that *S. indica* treatment altered the microbial community structure of tea plant leaves, which in turn suppressed fungal pathogens. However, whether *S. indica* confers drought tolerance in tea plants, and what the underlying mechanisms are, remains mysterious.

Here, we hypothesized that *S. indica* may exert a role in tea plants against drought stress. To explore this possibility, we integrated physiological, transcriptomic, and metabolomic approaches to investigate the effects of *S. indica* on the morphology, physiology, gene transcription, and metabolite generation of tea plants during different stages of drought stress. This study deepens the understanding of the regulatory mechanisms of *S. indica* in tea plants and would provide guiding strategies for crop drought tolerance improvement.

## 2. Results

### 2.1. S. indica Root Colonization and Phenotypic and Physiological Responses of S. indica-Treated Tea Plants Under Drought Stress

**Figure 1:**
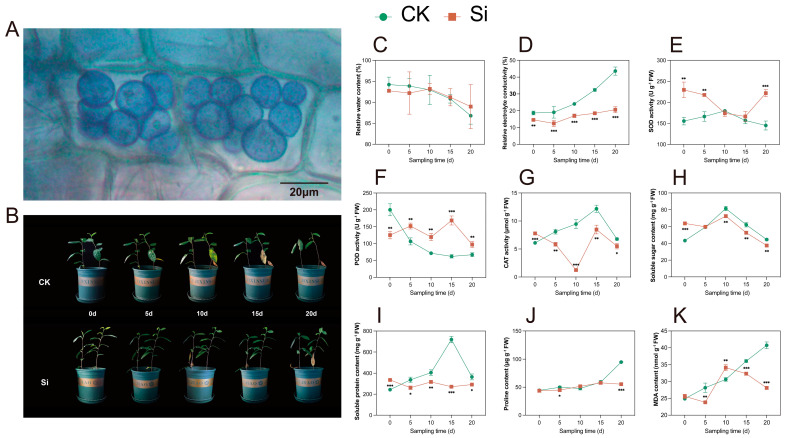
*S. indica* colonization and phenotypic and physiological traits of *S. indica*-treated and control tea plants under drought stress. (**A**) Tea plants root spores. (**B**) Phenotypic traits of *S. indica*-treated and control tea plants. (**C**) Relative water content of leaves. (**D**) Relative electrolyte conductivity of leaves. (**E**–**G**) Activities of superoxide dismutase (SOD), peroxidase (POD), and catalase (CAT). (**H**–**K**) Contents of soluble sugar, soluble protein, proline, and malondialdehyde (MDA). Data are expressed as mean ± standard deviation from three biological experiments. *, **, and *** indicate significant differences between Si and CK at the *p* < 0.05, *p* < 0.01, and *p* < 0.001 levels, respectively.

Following 30 days of co-culturing *S. indica* with tea plants, root cells were stained with trypan blue for observation. As in the colonization of other plants’ roots, *S. indica* was observed to colonize in the roots of tea plants, and the pear-shaped spores were gathered on the root cortical cells (Figure 1A).

After 5 d of drought stress, the bottom leaf color of both *S. indica*-inoculated (Si) and uninoculated (CK) tea plants turned yellow-green (Figure 1B). At 10 d, the CK group leaves browned and dropped, and the top buds wilted; most leaves abscised after 20 d. *S. indica*-treated tea plants delayed bud wilting until day 20, and fewer leaves abscised. The relative water content (RWC) of the leaves decreased as the duration of the drought treatment increased in both the Si and CK groups, and there was no significant difference (*p* ≥ 0.05) between the Si and the CK (Figure 1C). The relative electrolyte conductivity (REC) of the leaves increased as the duration of the drought treatment increased in both the Si and CK groups, and the Si showed a lower REC than the CK (Figure 1D).

Regarding antioxidant enzyme activities, SOD activity was higher in the Si than in the CK at 0, 5, and 20 d of drought treatment (Figure 1E). The Si exhibited lower POD activity and higher CAT activity compared with the CK at 0 d (Figure 1F,G); after 5 d, the POD activity of the tea plants inoculated with *S. indica* was higher than that of the CK, while the CAT activity of the tea plants inoculated with *S. indica* was lower than that of the CK. Regarding osmoregulatory substances, the soluble sugar and soluble protein contents were higher in the Si than in the CK at 0 d, and proline content was higher in the Si than in the CK at 20 d (Figure 1H–J). Notably, the changes in soluble protein and proline contents in the Si were much smaller than those in the CK. The Si group exhibited generally lower MDA levels compared with the CK group (Figure 1K). The Si group and the CK group showed significant differences in phenotype and physiological content at 10 and 20 days of drought treatment. Consequently, we collected leaf samples on days 10 and 20 for subsequent research.

### 2.2. Transcriptome Analysis of S. indica-Treated Tea Plants Under Drought Stress

To explore the molecular events in *S. indica*-treated tea plants under drought stress, RNA-seq was employed to investigate the differentially expressed genes (DEGs) between the Si and CK groups under drought conditions. A total of 100.78 Gb of clean data was obtained, and each sample yielded at least 7 Gb (Table S1). The Q30 and GC content percentages ranged from 96.57% to 97.00% and 45.11% to 45.87%, respectively. More than 88.27% of the clean reads were mapped to the reference genome. To validate the expression patterns of the genes, 16 DEGs were selected for quantitative real-time PCR (qRT-PCR) analysis (Figure S1). The expression trends of these DEGs were consistent with the fragments per kilobase of transcript per million fragments mapped (FPKM) values of the RNA-seq data. These DEGs were regulated by *S. indica* under drought conditions, suggesting not only the reliability of our transcriptome data but also that these selected genes may be important candidates for *S. indica* in modulating drought tolerance in tea plants.

**Figure 2:**
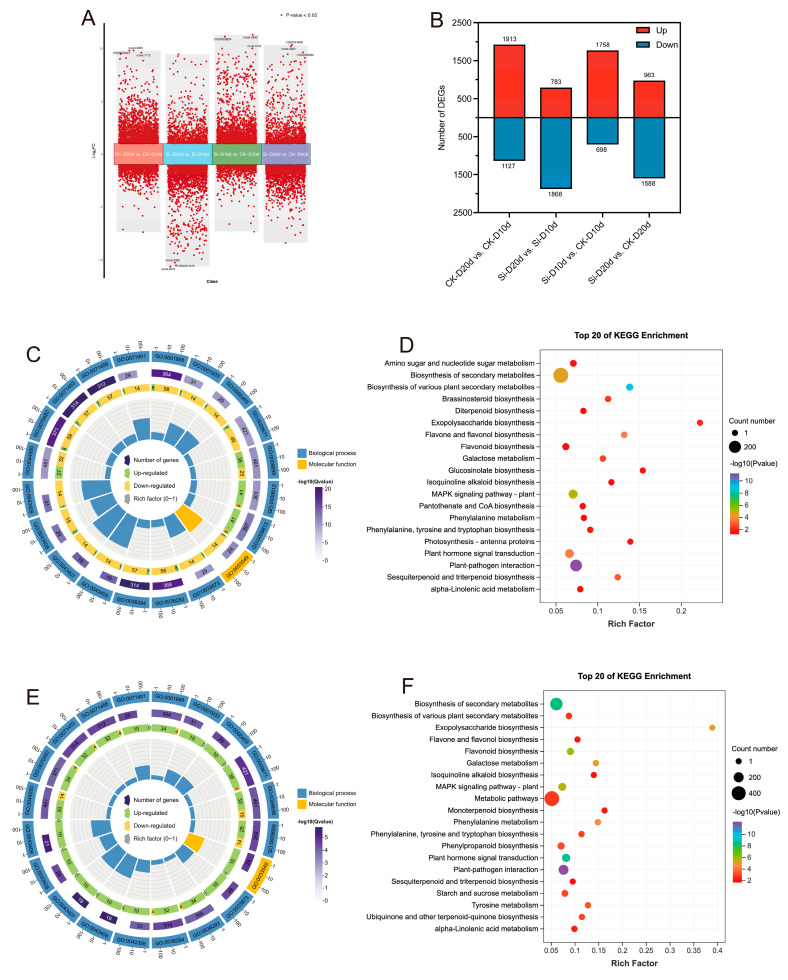
Transcriptome analysis of *S. indica*-treated and control tea plant samples under drought stress. (**A**) Volcano plot of differentially expressed genes (DEGs) in different comparison groups. (**B**) Number of DEGs in different comparison groups. (**C**) Top 20 GO terms enrichment analysis of DEGs in Si-D10d vs. CK-D10d. (**D**) Top 20 GO terms enrichment analysis of DEGs in Si-D20d vs. CK-D20d. (**E**) Top 20 KEGG pathways enrichment analysis of DEGs in Si-D10d vs. CK-D10d. (**F**) Top 20 KEGG pathways enrichment analysis of DEGs in Si-D20d vs. CK-D20d.

Principal component analysis (PCA) indicated that *S. indica* had pronounced effects on tea plants’ transcriptome (Figure S2). Pearson correlation coefficient (PCC) among the three replicate samples exceeded 0.98, indicating high reliability of the data (Figure S2). In the comparison analysis, 2456 DEGs (1758 upregulated and 698 downregulated) were identified in Si-D10d vs. CK-D10d, and 2551 DEGs (963 upregulated and 1588 downregulated) were identified in Si-D20d vs. CK-D20d (Figure 2A,B). To elucidate the biological functions of the candidate DEGs, Gene Ontology (GO) and Kyoto Encyclopedia of Genes and Genomes (KEGG) enrichment analyses were conducted (Figure 2C–F). Among the top 20 GO terms, DEGs from both groups were significantly (*p* < 0.05) enriched in biological processes (BPs) and molecular functions (MFs). The shared enriched terms included regulation of MAPK cascade (GO:0043408), cellular response to hypoxia (GO:0071456), phenylpropanoid metabolic process (GO:0009698), and secondary metabolite biosynthetic process (GO:0044550) (Figure 2C,E). The DEGs in Si-D10d vs. CK-D10d showed significant enrichment in phenylpropanoid biosynthetic process (GO:0009699) and negative regulation of phosphorylation (GO:0042326), and DEGs in Si-D20d vs. CK-D20d exhibited significant enrichment in flavonoid biosynthetic process (GO:0009813) and flavonoid metabolic process (GO:0009812). The DEGs in Si-D10d vs. CK-D10d and Si-D20d vs. CK-D20d were enriched in several common KEGG pathways. These included plant–pathogen interaction, plant hormone signal transduction, MAPK signaling pathway—plant, flavonoid biosynthesis, and galactose metabolism (Figure 2D,F). Additionally, DEGs in Si-D10d vs. CK-D10d were significantly enriched in brassinosteroid biosynthesis and amino sugar and nucleotide sugar metabolism, while DEGs in Si-D20d vs. CK-D20d were significantly enriched in tyrosine metabolism, starch and sucrose metabolism, and phenylpropanoid biosynthesis. These results suggested that *S. indica* could modulate complex gene expression patterns and biochemical pathways in tea plants under drought stress.

### 2.3. S. indica-Induced Genes Under Drought Stress

**Figure 3:**
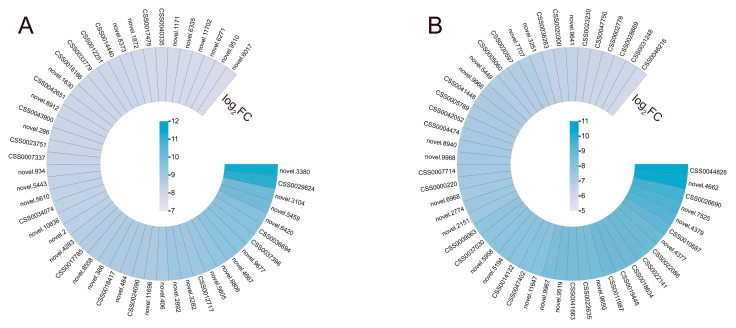
The changes of key DEGs of *S. indica*-induced in tea plant response to drought stress. (**A**) Heatmap of 50 DEGs with the highest log_2_FC in Si-D10d vs. CK-D10d. (**B**) Heatmap of 50 DEGs with the highest log_2_FC in Si-D20d vs. CK-D20d.

*S. indica* elicits particular alterations in gene expression in reaction to drought stress. The 50 DEGs with the highest log_2_ (fold change, FC) in both Si-D10d vs. CK-D10d and Si-D20d vs. CK-D20d were selected for study (Figure 3; Table S2). In the set of Si-D10d vs. CK-D10d, multiple drought stress-responsive genes were identified, including *heat shock 70 kDa protein* (*Hsp70*, novel.9677), *late embryogenesis abundant* (*LEA*, novel.5610), and genes related to stomatal closure and K^+^ transport (novel.9510 and novel.8912) (Figure 3A; Table S2). In the Si-D20d vs. CK-D20d set, eight *glutathione S-transferase* (*GST*) genes (CSS0022141, CSS0026690, CSS0018634, novel.4377, CSS0022086, CSS0005789, CSS0031248, and CSS0028669) and eight key structural genes associated with flavonoid biosynthesis, including one *leucoanthocyanidin reductase* (*LAR*, CSS0009063), one *anthocyanidin reductase* (*ANR*, CSS0041663), three *chalcone synthase* (*CHS*, CSS0007714, CSS0004474, and CSS0030597), two *anthocyanidin synthase* (*ANS*, CSS0010687 and CSS0046216), and one *flavonoid 3′,5′-hydroxylase* (*F3′5′H*, CSS0014132), were found (Figure 3B; Table S2). Moreover, two MYB family genes (CSS0005060 and CSS0000220) were also identified. These findings demonstrated that *S. indica* could stimulate transcription factors (TFs) and genes involved in antioxidant defense, stomatal regulation, and ion transport.

### 2.4. Identification of S. indica-Activated Transcription Factors Under Drought Stress

**Figure 4:**
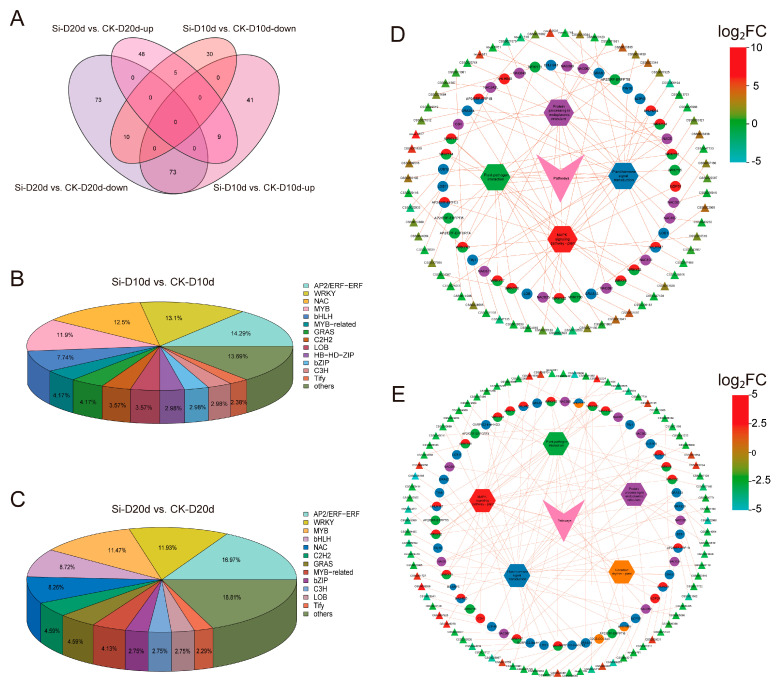
Transcription factors (TFs) analysis of *S. indica*-treated tea plant samples under drought stress. (**A**) Venn analysis of TFs in Si-D10d vs. CK-D10d and Si-D20d vs. CK-D20d. (**B**) Classification of identified TFs in Si-D10d vs. CK-D10d. (**C**) Classification of identified TFs in Si-D20d vs. CK-D20d. (**D**) Co-expression network of TFs and genes in significantly enriched KEGG pathways in Si-D10d vs. CK-D10d. (**E**) Co-expression network of TFs and genes in the significantly enriched KEGG pathways in Si-D20d vs. CK-D20d. Hexagons represent KEGG pathways, pies represent TFs, and triangles represent genes, with triangle colors shaded from light to dark based on the log_2_FC.

To clarify the regulatory mechanisms of drought responses triggered by *S. indica* in tea plants, we performed a comprehensive analysis of transcripts encoding TFs. In Si-D10d vs. CK-D10d and Si-D20d vs. CK-D20d, 168 (123 upregulated and 45 downregulated) and 218 (62 upregulated and 156 downregulated) TFs were identified, respectively (Figure 4A; Tables S3 and S4). These TFs were derived from many families, including AP2/ERFs, MYBs, WRKYs, NACs, and bHLHs (Figure 4B,C). Nine *TFs* (CSS0033018, CSS0021675, CSS0005060, CSS0007925, CSS0016632, CSS0018453, CSS0001246, CSS0032956, and CSS0036919) were consistently upregulated in both comparison groups (Tables S3 and S4). Further, KEGG enrichment analysis revealed that these TFs were significantly enriched in pathways such as plant hormone signal transduction, MAPK signaling pathway—plant, plant–pathogen interaction, and protein processing in the endoplasmic reticulum (Figure S3).

We constructed a co-expression network of TFs and genes within the significantly enriched KEGG pathways (Figure 4D,E). In the plant hormone signal transduction pathway, TFs such as *LOB11* (*ARF*, CSS0010889), *GRAS13* (*DELLA*, CSS0033997), *AP2*/*ERF-ERF1B* (*ERF1*, CSS0021082), *Tify10* (*JAZ*, CSS0034430), *bHLH147* (*MYC2*, CSS0047466), and *bZIP46* (*TGA*, CSS0007925) regulated auxin, gibberellin (GA), ethylene (ETH), jasmonic acid (JA), and salicylic acid (SA) signaling, respectively (Figure 4D,E; Tables S3 and S4). Genes in the MAPK signaling pathway—plant and plant–pathogen interaction pathways were primarily regulated by WRKY family members, including *WRKY33* (CSS0037160 and CSS0013638), *WRKY40* (CSS0028565 and CSS0029116), and *WRKY22* (CSS0021156). *NAC090* (CSS0010836 and CSS0022987), *NAC062* (CSS0026968 and CSS0019384), and *NAC6* (CSS0026968 and CSS0025009) played crucial roles in protein processing in the endoplasmic reticulum pathway. Interestingly, the expression levels of these TF genes, excluding CSS0007925, were increased in Si-D10d vs. CK-D10d but decreased in Si-D20d vs. CK-D20d. These *S. indica*-activated TFs may serve as potential regulators in the drought adaptation of tea plants.

### 2.5. Weighted Gene Co-Expression Network Analysis (WGCNA)

**Figure 5:**
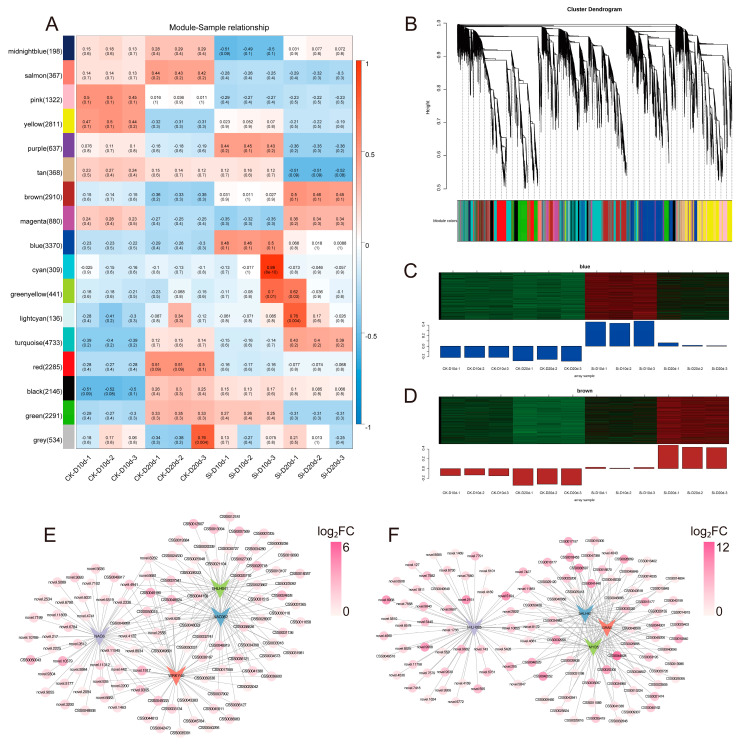
Weighted gene co-expression network analysis (WGCNA) of *S. indica*-treated and control tea plant samples under drought stress for 10 and 20 days. (**A**) Relationship among co-expression modules and samples. Numbers in brackets next to the modules indicate the number of genes within that module. The heatmap displays the correlation between co-expression modules (*y*-axis) and samples (*x*-axis), where blue indicates negative correlation and red indicates positive correlation. *p*-values are shown in parentheses. (**B**) Hierarchical clustering tree of 17 co-expressed gene modules. (**C**) Gene expression profiles of the blue module across different samples. (**D**) Gene expression profiles of the brown module across different samples. The upper section presents a clustered heatmap of genes within the module, where red indicates high expression levels and green indicates low expression levels. The lower section displays the expression pattern of the module eigengene across different samples. (**E**) Gene regulatory network of the blue module. (**F**) Gene regulatory network of the brown module.

WGCNA was conducted on 25,738 genes based on gene expression patterns, generating 17 modules (Figure 5A,B). The blue and brown modules, which were strongly positively correlated with Si-D10d and Si-D20d, respectively, exhibited increased FPKM in *S. indica*-treated tea plants compared with the controls (Figure 5A–D). Therefore, these two modules were selected for further analysis.

In the blue module (288 DEGs with 4 *TFs*) and the brown module (486 DEGs with 36 *TFs*), DEGs were upregulated in Si-D10d vs. CK-D10d and Si-D20d vs. CK-D20d, respectively (Tables S5 and S6). Due to KEGG annotation, DEGs in the blue module were predominantly associated with the regulation of biological processes, specifically plant–pathogen interaction and MAPK signaling pathway—plant (Figure S4; Table S7). DEGs in the brown module primarily linked to metabolic processes, particularly flavonoid biosynthesis and starch and sucrose metabolism (Figure S4; Table S8), which were consistent with previous DEGs enrichment results.

Drought-responsive DEGs related to signal transduction, abscisic acid (ABA) biosynthesis and metabolism, as well as water transport and stress protection, were identified. For example, we found *mitogen-activated protein kinase 13* (*MPK13*, CSS0019707), *9-cis-epoxycarotenoid dioxygenase NCED* (*NCED*, CSS0033791), and *peroxidase* (*PER*, CSS0037341) in the blue module (Table S5). In the brown module, we recognized *sucrose non-fermenting 1-related protein kinase 2* (*SnRK2*, CSS0019760) and *mitogen-activated protein kinase kinase kinase 20* (*M3K20*, CSS0034980), which participate in the MAPK signaling, as well as *abscisic acid 8′-hydroxylase 2* (*ABAH2*, CSS0002199), a cytochrome P450 family member that modulates ABA degradation (Table S6). In addition, the brown module contained the *aquaporin PIP2-7* (*PIP2-7*, CSS0001968) and *LEA65* (CSS0031198) genes, along with multiple POD-encoding genes (CSS0028286, CSS0026112, and CSS0025824). Key genes involved in the flavonoid biosynthesis pathway, such as *ANS*, *ANR*, and *LAR*, were identified in both modules. The gene regulatory network map depicted the association of four *TFs* with the highest connectivity in the blue and brown modules, respectively (Figure 5E,F). These pathways, TFs, and genes may play significant roles in the drought tolerance regulated by *S. indica*.

### 2.6. Metabolome Profile of S. indica-Treated Tea Plants Under Drought Stress

**Figure 6:**
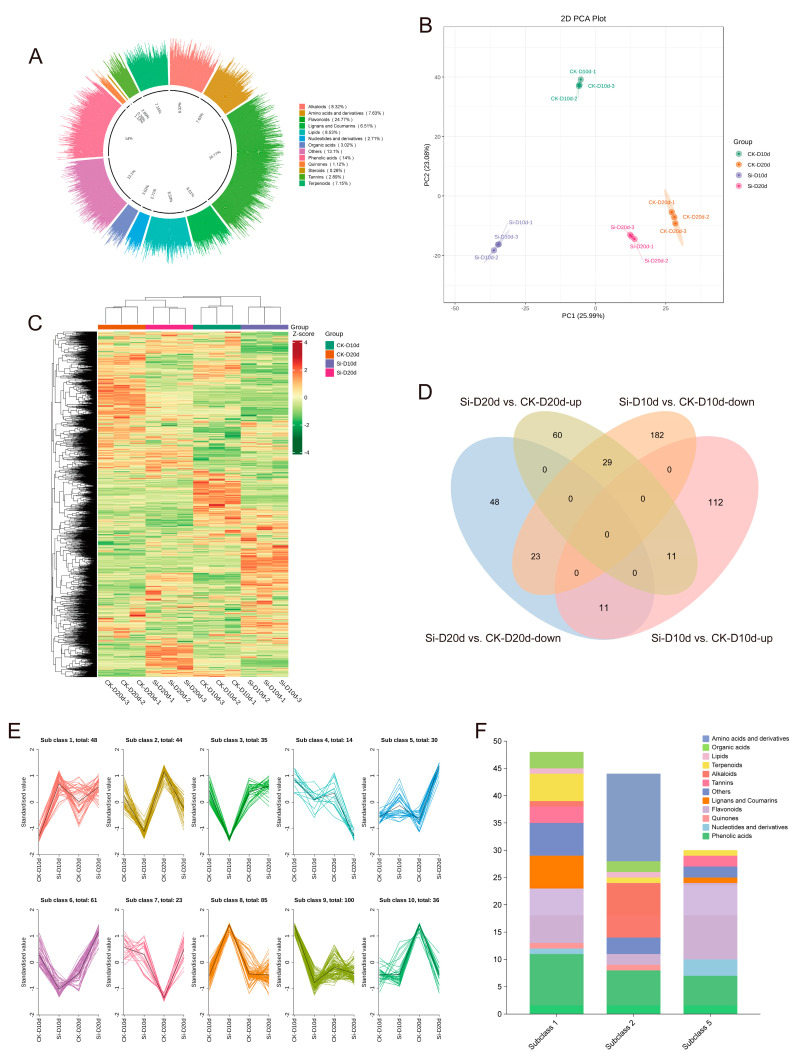
Metabolomic analysis of *S. indica*-treated and control tea plant samples under drought stress. (**A**) Categories of 2321 metabolites. (**B**) PCA of metabolites. (**C**) Hierarchical clustering analysis of metabolites in 12 samples. (**D**) Venn analysis of differentially accumulated metabolites (DAMs) in Si-D10d vs. CK-D10d and Si-D20d vs. CK-D20d. (**E**) K-Means analysis. (**F**) Metabolites classification in subgroups 1, 2, and 5.

To evaluate the metabolite composition of *S. indica*-treated tea plants under drought stress, tea plant leaves were subjected to a broadly targeted metabolomics analysis. A total of 2321 metabolites were identified, comprising flavonoids (24.77%), alkaloids (8.32%), phenolic acids (14%), lipids (8.53%), lignans and coumarins (6.51%), terpenoids (7.15%), nucleotides and derivatives (2.71%), amino acids and derivatives (7.63%), organic acids (3.02%), tannins (2.89%), quinones (1.12%), steroids (0.26%), and other (13.1%) compounds (Figure 6A). PCA revealed that PC1 and PC2 together accounted for 49.07% of the variance among samples, clearly separating the different treatments (Figure 6B). The hierarchical clustering heatmap showed that samples exposed to 20 d of drought stress clustered together, and the metabolic changes in CK-D10d were more similar to those in CK-D20d and Si-D20d compared to Si-D10d (Figure 6C). This indicated significant alterations in the metabolite profiles of tea plant leaves inoculated with *S. indica* under drought stress.

In Si-D10d vs. CK-D10d and Si-D20d vs. CK-D20d, 368 (134 upregulated and 234 downregulated) and 182 (100 upregulated and 82 downregulated) differentially accumulated metabolites (DAMs) were identified, respectively (Figure S5; Tables S9 and S10). As shown in Figure 6D, 11 upregulated and 23 downregulated DAMs were shared between Si-D10d vs. CK-D10d and Si-D20d vs. CK-D20d. The eleven upregulated DAMs primarily included seven flavonoids, such as apigenin-6-C-arabinoside-4′-O-rhamnoside, tangeretin, and nobiletin, as well as three phenolic acids, including 5′-hydroxyhexahydrocurcumin and 3,4-dihydroxybenzoic acid (Table S11). The 23 shared downregulated DAMs mainly consisted of amino acids and derivatives (10) and alkaloids (7), including N-palmitoylglycine, N6-acetyl-L-lysine, L-arginine, N-benzylmethylene isomethylamine, and indoline (Table S11).

K-Means clustering analysis was performed on the DAMs identified in Si-D10d vs. CK-D10d and Si-D20d vs. CK-D20d to investigate the relative changes in metabolite content across different groups (Figure 6E). The DAMs were divided into ten subclasses, where the relative content of metabolites in subgroups 1 and 5 increased, whereas that in subclass 2 decreased after *S. indica* treatment. DAMs in subclass 1 were predominantly phenolic acids and flavonoids; those in subclass 5 were mainly flavonoids; and, in subclass 2, the primary metabolites were amino acids and derivatives and alkaloids (Figure 6F). The results indicated that *S. indica* treatment reduced the content of amino acids and derivatives and alkaloids in tea plants while promoting the accumulation of flavonoids and phenolic acids under drought stress.

### 2.7. Integrative Analysis of Transcriptome and Metabolome

**Figure 7:**
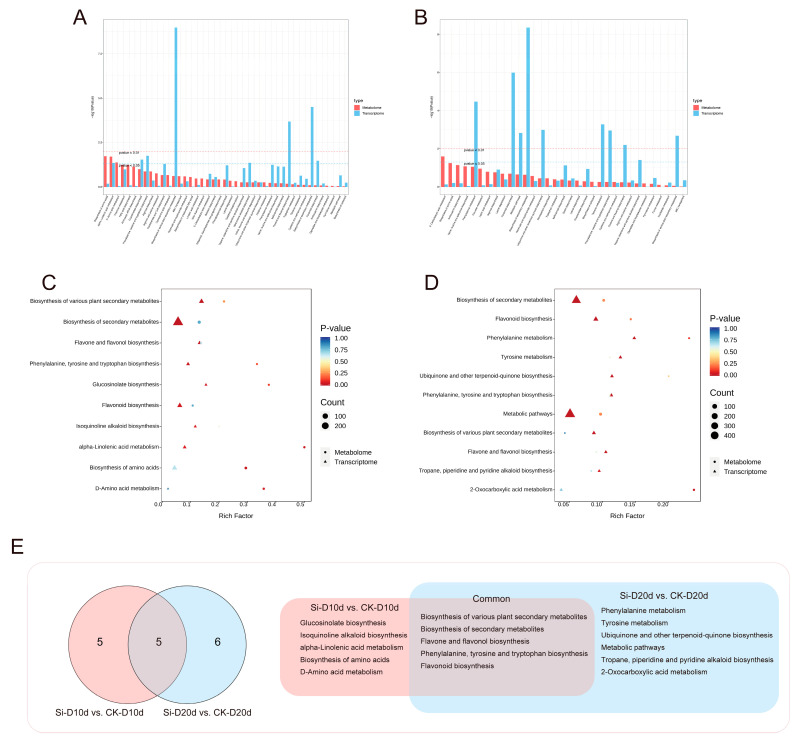
Comprehensive analysis of transcriptome and metabolome of *S. indica*-treated and control tea plant samples under drought stress for 10 and 20 days. (**A**) KEGG analysis of the transcriptome and metabolome in Si-D10d vs. CK-D10d. (**B**) KEGG analysis of transcriptome and metabolome in Si-D20d vs. CK-D20d. (**C**) Significantly enriched KEGG pathways in Si-D10d vs. CK-D10d. (**D**) Significantly enriched KEGG pathways in Si-D20d vs. CK-D20d. (**E**) Venn diagram of co-annotated KEGG pathways that were significantly enriched in either the metabolome or transcriptome of Si-D10d vs. CK-D10d and Si-D20d vs. CK-D20d.

Joint transcriptome and metabolomic analyses were conducted to identify key KEGG pathways influenced by *S. indica* in response to drought stress. KEGG analysis revealed that DEGs and DAMs in both Si-D10d vs. CK-D10d and Si-D20d vs. CK-D20d were enriched mainly in the biosynthesis and metabolites of amino acids and flavonoids (Figure 7A,B). By selecting significant (*p* < 0.05) KEGG pathways from at least one omics dataset within each of the Si-D10d vs. CK-D10d and Si-D20d vs. CK-D20d, a total of five KEGG pathways were shared to both groups (Figure 7C–E). The pathways included the biosynthesis of secondary metabolites, flavonoid biosynthesis, phenylalanine, tyrosine, and tryptophan biosynthesis, biosynthesis of various plant secondary metabolites, and flavone and flavonol biosynthesis. Comprehensive analysis emphasized the particularly crucial role of the flavonoid biosynthesis pathway in the *S. indica*-induced drought stress response.

### 2.8. Analysis of Genes and Metabolites Related to Flavonoid Synthesis Pathways

**Figure 8:**
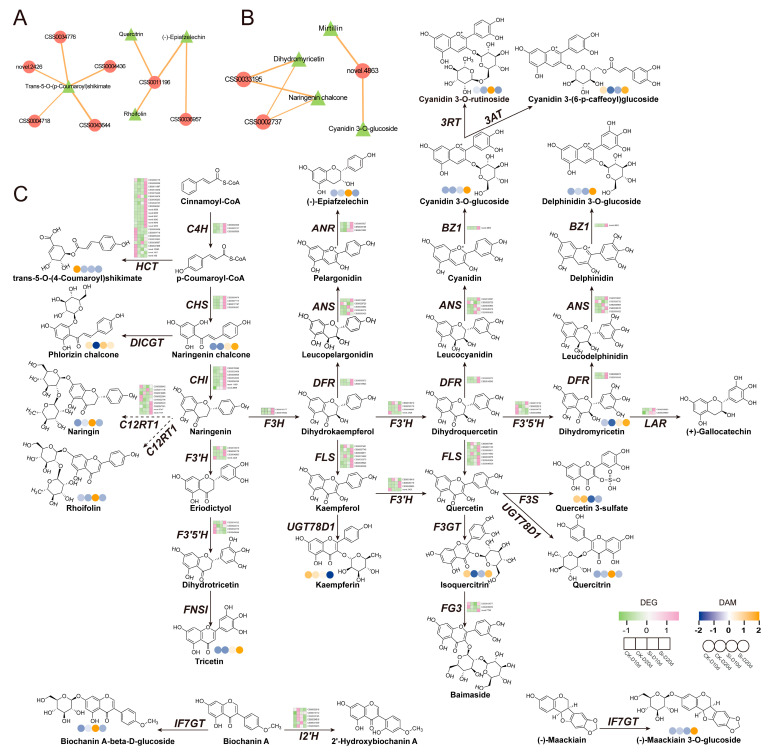
Co-expression analysis of DEGs and DAMs involved in flavonoid biosynthesis. (**A**) Interaction network of DEGs and DAMs involved in flavonoid biosynthesis in Si-D10d vs. CK-D10d. (**B**) Interaction network of DEGs and DAMs involved in flavonoid biosynthesis in Si-D20d vs. CK-D20d. Solid lines indicate positive correlations, dashed lines indicate negative correlations, and the thickness of the lines represents the strength of the correlation. (**C**) Expression profiles of DEGs and DAMs involved in flavonoid biosynthesis. *C4H*, cinnamate 4-hydroxylase; *HCT*, shikimate O-hydroxycinnamoyltransferase; *CHS*, chalcone synthase; *DICGT*, chalcononaringenin 2′-O-glucosyltransferase; *CHI*, chalcone isomerase; *C12RT1*, flavanone 7-O-glucoside 2″-O-beta-L-rhamnosyltransferase; *F3H*, flavanone 3-hydroxylase; *F3′H*, flavonoid 3′-hydroxylase; *F3′5′H*, flavonoid 3′,5′-hydroxylase; *FNS1*, flavone synthase I; *FLS*, flavonol synthase; *UGT78D1*, flavonol-3-O-rhamnosyltransferase; *DFR*, dihydroflavonol 4-reducatse; *ANS*, anthocyanidin synthase; *ANR*, anthocyanidin reductase; *LAR*, leucoanthocyanidin reductase; *BZ1*, anthocyanidin 3-O-glucosyltransferase; *3AT*, anthocyanidin 3-O-glucoside 6″-O-acyltransferase; *F3GT*, flavonol 3-O-glucosyltransferase; *FG3*, flavonol-3-O-glucoside; *F3S*, flavonol 3-sulfotransferase; *IF7GT*, isoflavone 7-O-glucosyltransferase; *I2′H*, isoflavone 2′-hydroxylase.

To investigate the impact of *S. indica* on genes and metabolites related to flavonoid biosynthesis in tea plants subjected to drought stress, DEGs and DAMs with |Pearson correlation coefficient| > 0.8 and *p* < 0.05 from the flavonoid biosynthesis were selected for network diagram construction (Figure 8A,B). In Si-D10d vs. CK-D10d, CSS0011196 showed positive correlations with (-)-epiafzelechin, quercitrin, and rhoifolin, encoding flavanone 7-O-glucoside 2″-O-beta-L-rhamnosyltransferase (C12RT1) (Figure 8A). Additionally, genes (CSS0043644, CSS0034776, CSS0004436, novel.2426, and CSS0004718) positively correlated with trans-5-O-(p-coumaroyl)shikimate, encoding F3′5′H, flavonoid 3′-hydroxylase (F3′H), and shikimate O-hydroxycinnamoyltransferase (HCT). In Si-D20d vs. CK-D20d, CSS0002737, CSS0033195, and novel.4863 encoded cinnamate 4-hydroxylase (C4H), ANR, and anthocyanidin 3-O-glucosyltransferase (BZ1), respectively (Figure 8B).

We mapped metabolite levels and gene expression profiles onto the flavonoid biosynthesis pathway (Figure 8C). RNA-seq analysis revealed that DEGs encoding key enzymes in flavonoid biosynthesis—*C4H*, *F3H*, *ANR*, *CHS*, *LAR*, and dihydroflavonol 4-reducatse (*DFR*)—were upregulated in both Si-D10d vs. CK-D10d and Si-D20d vs. CK-D20d. Notably, most of these DEGs had a higher fold change in Si-D20d vs. CK-D20d than in Si-D10d vs. CK-D10d. Metabolomic analysis indicated substantial accumulation of quercetin-3-O-rhamnoside, rhoifolin, and (-)-epiafzelechin in Si-D10d vs. CK-D10d and significant accumulation of cyanidin-3-O-glucoside, delphinidin-3-O-glucoside, and dihydromyricetin in Si-D20d vs. CK-D20d. These results indicated that *S. indica* treatment induced significant dynamic changes in genes and metabolites related to flavonoid biosynthesis in tea plants under varying degrees of drought stress, thereby enhancing tea plants’ adaptation to environmental changes.

## 3. Discussion

Limited and diminishing renewable freshwater supplies for agricultural irrigation necessitate urgent enhancements in crop drought tolerance to maintain agricultural productivity [36]. *S. indica* serves as a biological tool to improve plant drought tolerance and appears to be an innovative approach towards sustainable agriculture [37]. Although *S. indica* has demonstrated the ability to enhance drought resistance in other host plants [22,32], studies on its interaction with tea plants under drought conditions remain scarce.

The parameters of RWC and REC were closely related to drought resistance. RWC reflects the leaf water status, and REC estimates the leaf cell membrane stability in plants [38]. In this study, the RWC decreased and the REC increased with drought time in both Si and CK groups (Figure 1). Notably, the REC was lower in the Si than in the CK, indicating that *S. indica* inoculation enhances the protective mechanism of tea plant leaf cell membranes under drought conditions. Based on the phenotypic changes, and RWC and REC measurements, we found that *S. indica* could alleviate the negative impacts of drought stress on tea plants.

Drought is known to disrupt the balance between antioxidants and ROS levels, leading to oxidative stress in plant cells. Plants can utilize the protective enzyme system, including SOD, POD, and CAT, to prevent ROS accumulation [39]. Previous studies on *Chinese cabbage* by Sun et al. [25] and on wheat by Yaghoubian et al. [24] have demonstrated that inoculation with *S. indica* significantly increased CAT activity under drought conditions. In contrast, our study showed that CAT activity in *S. indica*-inoculated plants was lower than that in the CK group under drought stress (Figure 1), a finding consistent with the results reported by Hosseini et al. [18] in wheat. These observations collectively suggested that the impacts of *S. indica* on antioxidant enzyme activities are variable in plants. These variations could be due to differences in experimental conditions and drought stress levels, genetic and physiological differences among plant species, strain-specific effects of *S. indica*, distinct interaction dynamics between *S. indica* and the host plants, and interactions of *S. indica* with other microorganisms. On the other hand, *S. indica* inoculation increased SOD and POD activities in tea plants compared with the CK under drought stress, which was similar to previous reports [33,40]. Additionally, our work discovered numerous POD-encoding genes (CSS0037341, CSS0028286, CSS0026112, and CSS0025824) from the DEGs sets, indicating that POD may function as a key antioxidant enzyme in tea plants’ response to *S. indica*-mediated drought resistance. Drought also induces osmotic stress in plants, resulting in cell dehydration and ion imbalance [8]. To adapt to osmotic stress, plants accumulate compatible solutes like soluble sugar, soluble protein, and proline [41]. In this study, *S. indica* inoculation significantly reduced the content of soluble sugar, soluble protein, and proline compared with the CK under drought conditions, which was somewhat different from studies in other plants [40,42]. However, the levels of these osmolytes demonstrated increased stability after *S. indica* inoculation under drought stress. This suggested that *S. indica* inoculation activates drought-escape mechanisms in tea plants, reducing the need to accumulate osmoprotectants in response to drought. MDA is a biomarker of oxidative damage, primarily generated through ROS-induced degradation of polyunsaturated fatty acids [43]. In this study, inoculation with *S. indica* resulted in lower MDA levels during all stages of drought stress except at 10 d, indicating that *S. indica* inoculation mitigates oxidative damage in tea plants under drought conditions, which was similar to the results in other plants [21]. In summary, we could conclude that *S. indica* inoculation enhanced drought tolerance in tea plants through reducing oxidative damage, modulating antioxidant enzyme activities, and stabilizing osmolyte levels.

Flavonoids are known to play an important role in plant growth, environmental adaptation, and defense against adversity stress, besides possessing medicinal and nutritional value [44]. Among various environmental factors affecting plant growth, drought is the most widespread [45]. The flavonoid pathway was reported to respond to water deficit stress in multiple plants, including *Populus* [6], *Hordeum vulgare* [46], maize [7], and *Ligustrum vulgare* [47]. However, the changes in flavonoid profiles of *S. indica*-treated plants under drought stress remain elusive. Although Tyagi et al. [42] reported an increase in flavonoid content in the presence of *S. indica* during drought, a comprehensive understanding of the underlying reshaping processes is still lacking. In this study, metabolomic analysis revealed that *S. indica*-inoculated tea plants accumulated higher levels of flavonoids than uninoculated plants during drought stress (Figure 8). The Venn diagram showed 11 overlapping upregulated metabolites in Si-D10d vs. CK-D10d and Si-D20d vs. CK-D20d. Among these, seven metabolites were identified as flavonoids, including five polymethoxyflavones (PMFs), one apigenin glycoside derivative, and one vitexin glycoside derivative (Figure 6; Table S11). PMFs are synthesized from flavonoids through methylation modifications and exhibit rich biological activities, effectively scavenging ROS [48]. Among the identified DAMs, there were many flavonoid glycosides catalyzed by UDP-dependent glycosyltransferases (UGTs) (Tables S9 and S10). Glycosyltransferases enhance the water solubility and stability of flavonoids by catalyzing the transfer of glycosyl groups onto flavonoid molecules, thereby improving their bioavailability and antioxidant activity [49]. This investigation revealed that *UGT1* and *UGT2* genes were enriched in WGCNA results (Tables S5 and S6). Recent studies have reported that glycosyltransferase genes can enhance the drought resistance of tea plants [50,51]. We found that *S. indica*-inoculated tea plants showed dynamic flavonoid responses under drought conditions. *S. indica*-inoculated tea plants accumulated more flavonols (quercetin-3-O-rhamnoside) and flavanols ((-)-epiafzelechin) after 10 d of drought treatment (Figure 8), at which point *S. indica* enhanced the drought adaptability of tea plants by regulating stomatal movements [7,52]. Anthocyanins (cyanidin-3-O-glucoside and delphinidin-3-O-glucoside) were the main upregulated metabolites at 20 d, contributing to osmotic adjustment and scavenging excess ROS to cope with prolonged drought [53]. These results showed that flavonoids might play a vital role in *S. indica*-inoculated tea plants response to drought stress.

In this study, we reported that the expression of *C4H* and *CHS* was upregulated (Figure 8). The results were in agreement with the report on *Capsicum annuum* under drought conditions [54]. Naringenin, a general precursor of flavonoids, is converted to dihydroflavonol under the catalysis of F3H, which is the key enzyme in dihydroflavonol synthesis [55]. *F3H* transcripts were clearly enhanced by water deficit in grape berries [56] and *Reaumuria soongorica* [57]. After inoculation with *S. indica*, we observed an increased expression of *F3H* under drought stress. DFR, LAR, and ANR are the key enzymes in anthocyanin biosynthesis [58]. Drought exposure triggers the transcription of a series of anthocyanin biosynthesis genes in many plants [59,60]. In this study, *S. indica* inoculation upregulated the transcription levels of *DFR*, *LAR*, and *ANR* under drought stress, with the expression levels of these genes increasing as drought stress intensified. These findings suggested that these genes may mediate the adaptive response of tea plants treated with *S. indica* to drought stress.

In this study, we also analyzed the key TFs involved in the *S. indica*-induced drought resistance in tea plants (Figure 4). Most TFs were clustered into AP2/ERF, MYB, WRKY, NAC, and bHLH families, and several TFs have been reported to participate in tea plant response to drought stress [61,62]. Notably, we found that the expression of *DREB3* (CSS0000024) was significantly downregulated in Si-D10d vs. CK-D10d but significantly upregulated in Si-D20d vs. CK-D20d; *DREB1D* (CSS0021954), *DREB2A* (novel.1407), and *DREB2C* (CSS0010594) were significantly upregulated in Si-D10d vs. CK-D10d and significantly downregulated in Si-D20d vs. CK-D20d (Tables S3 and S4). *CsDREB1D* has been reported to enhance drought stress resistance through overexpression in *Arabidopsis thaliana* [63], indicating that *DREB*-mediated drought stress response plays an important role in *S. indica*-induced drought tolerance. Among the upregulated TFs, nine were commonly upregulated in both Si-D10d vs. CK-D10d and Si-D20d vs. CK-D20d, six of which belonged to the MYBs. It has been well recognized that the MYB family of TFs were key regulators of flavonoid biosynthesis [64], which is a crucial component of plant responses to environmental stresses [65]. Therefore, MYB-mediated flavonoid synthesis plays an important role in the drought response induced by *S. indica*.

## 4. Materials and Methods

### 4.1. Fungal Inoculation, Plant Materials, and Drought Treatment

*S. indica* inoculum preparation was performed according to Cheng et al. [66]. Briefly, three 5 × 5 cm agar blocks containing *S. indica* were incubated in a triangular flask filled with 100 mL of sterile potato dextrose broth (PDB) medium at 28 °C and 200 rpm in the dark for 3–5 d, after which the resulting fermentation broth was diluted threefold with sterile water to prepare the inoculum [67,68].

Tea plant (*Camellia sinensis* cv. Fudingdabaicha) seedlings were obtained from Nanjing Yarun Tea Co., Ltd. (Nanjing, China). Healthy, uniformly growing one-year-old clonal tea plant seedlings were selected as experimental materials and transplanted into plastic pots (diameter 20 cm, height 22 cm) filled with a mixture of peat soil and vermiculite (3:1, *v*/*v*). These seedlings were maintained in a growth chamber at a temperature of 25 ± 2 °C, 70% relative humidity, and a 16/8 h light/dark cycle with 220 μmol m^−2^ s^−1^ light intensity. Before transplanting the seedlings, the roots of half of the seedlings were immersed in the *S. indica* inoculum solution (Si) for 6 h, and the other half were immersed in sterile water (CK) for 6 h. Subsequently, each tea plant was irrigated with 50 mL of the inoculum (Si) or sterile water (CK) every five days for a total of five applications. The Si group and the CK group were additionally supplied with the same amount of water every three days to maintain field production capacity.

Thirty days after *S. indica* inoculation, the root colonization status was evaluated by applying the staining procedure described by Rai et al. [69]. After confirming the colonization of *S. indica*, soil moisture content was measured using an SN-3002-TRREC-N01 Moisture Meter (Shandong VEMSEE Technology Co., Ltd., Jinan, China), and drought treatment was implemented by controlling soil moisture. The tea plants were fully watered at 9:00–10:00 a.m. to bring soil moisture to 80%. Thereafter, soil moisture level was measured at 9:00 a.m. daily for timely monitoring and water supplementation. When soil moisture dropped to 70 ± 3%, the third and fourth functional leaves from the top of tea seedlings were randomly collected to determine the RWC and REC of the leaves. Meanwhile, the first two mature leaves from the top were collected, immediately placed in liquid nitrogen, and stored at −80 °C for subsequent analysis. This day was recorded as day 0 (D0d) of drought stress. The second sampling was conducted when soil moisture further declined to 25 ± 3% by withholding water supply, and this day was recorded as day 5 (D5d) of drought stress. Soil moisture content was maintained at 25 ± 3%, and samples were taken every five days, which were recorded as days 10 (D10d), 15 (D15d), and 20 (D20d) of drought stress, respectively.

The experiment was designed based on a completely randomized design with two factors, including two fungi treatments (inoculated and non-inoculated plants) and five levels of drought treatments (0, 5, 10, 15, and 20 d of drought treatment). A total of ten treatment groups were established, Si-D0d, Si-D5d, Si-D10d, Si-D15d, Si-D20d, CK-D0d, CK-D5d, CK-D10d, CK-D15d, and CK-D20d, with three biological replicates for each treatment group.

### 4.2. Determination of Relative Water Content and Relative Electrolyte Conductivity of Leaves

The RWC and REC were measured for samples from both the Si group and CK group subjected to drought treatment for 0, 5, 10, 15, and 20 d, with three biological replicates and three technical replicates for each sample. The RWC of the leaves was determined in accordance with the method described by Yue et al. [70]. Freshly collected leaves were immediately weighed to obtain the fresh weight (FW). The leaves were then fully immersed in deionized water at room temperature for 12 h and gently blotted dry with filter paper to measure hydrated weight (HW). Subsequently, the leaves were oven-dried at 80 °C for 24 h, and the dry weight (DW) was recorded. The RWC was calculated using the following formula:

$$RWC=\frac{FW-DW}{HW-DW}\times 100$$

The REC was measured in line with the procedure described by Wang et al. [71]. Leaf tissue (0.3 g) was placed in distilled water (20 mL) and subjected to vacuum treatment. The samples were then shaken at 200 rpm and 25 °C for 2 h, after which the initial conductivity (R1) of the solution was measured at 25 °C using a HZPD-T503 Conductivity Meter (HUAZHI Electronic Technology Co., Ltd., Putian, China). Subsequently, the solution was boiled at 100 °C for 20 min to fully lyse the cell walls, cooled to 25 °C, and the final conductivity (R2) was recorded. The REC was calculated as follows:

$$REC=\frac{R1}{R2}\times 100$$

### 4.3. Determination of Biochemical Features

The biochemical features were determined for samples from both the Si group and CK group subjected to drought treatment for 0, 5, 10, 15, and 20 days, with three biological replicates and three technical replicates for each sample. A 0.1 g sample was ground into powder using liquid nitrogen, mixed with 1 mL of phosphate buffer (100 mM, pH = 7.8), and homogenized on an ice bath. The mixture was subsequently centrifuged at 4 °C and 12,000 rpm for 10 min, and the supernatant was collected for SOD, POD, and CAT activities and MDA, and soluble protein contents assays. Then, 0.1 g of frozen leaves was homogenized in 1 mL of distilled water and then placed in a 95 °C water bath for 10 min. After cooling, the mixture was centrifuged at 25 °C and 8000 rpm for 10 min. Subsequently, 0.1 mL of the supernatant was transferred into a 1.5 mL centrifuge tube, and 0.9 mL of distilled water was added to prepare the soluble sugar assay mixture. Then, 0.1 g of leaves mixed with 1 mL 3% sulfosalicylic acid were shaken in a 95 °C water bath for 10 min. The solution was then centrifuged at 10,000 rpm and 25 °C for 10 min, and the supernatant was collected after cooling as the proline extract.

SOD, POD, and CAT activities were measured using the water-soluble tetrazolium salt 8 (WST-8) method (Kit No. SOD-1-W) [72], guaiacol method (Kit No. POD-1-Y) [73], and ammonium molybdate colorimetric method (Kit No. CAT-1-W) [73], respectively. The contents of soluble sugar, soluble protein, proline, and MDA were assayed using the anthrone colorimetric method (Kit No. KT-1-Y) [74], bicinchoninic acid method (Kit No. BCAP-1-W) [75], acidic ninhydrin method (Kit No. PRO-1-Y) [74], and thiobarbituric acid method (Kit No. MDA-1-Y) [74], respectively. The biochemical indicators were measured using the corresponding kits from Suzhou Keming Biotechnology Co., Ltd. (Suzhou, China), according to the manufacturer’s instructions.

### 4.4. Transcriptome Sequencing

Leaf samples from tea plants of the Si group and the CK group subjected to drought treatment for 10 and 20 d were used for RNA-seq. Total RNA was extracted from the leaves of the tea plants using ethanol precipitation methods and CTAB-PBIOZOL, followed by assessment of RNA concentration and integrity with a Qubit 4.0 Fluorometer (Thermo Fisher Scientific, Waltham, MA, USA), and a Qsep400 Bioanalyzer (Bioptic, Inc., Hangzhou, China). mRNAs were enriched using Oligo(dT) magnetic beads, leveraging the structural characteristic that most eukaryotic mRNAs possess polyA tails. Sequencing libraries were generated using the NEBNext Ultra RNA Library Prep Kit for Illumina (New England Biolabs, Ipswich, MA, USA), and the resulting cDNA libraries were sequenced on the Illumina platform by Metware Biotechnology Co., Ltd. (Wuhan, China).

To obtain clean reads, raw data were filtered using fastp 0.23.2, and clean reads were then mapped to the *Camellia sinensis* cv. Shuchazao genome [76] using HISAT 2.2.1. Relative expression levels of transcripts were quantified and normalized based on fragments per kilobase of transcript per million fragments mapped (FPKM). DESeq2 1.22.1 was used for library size normalization, dispersion estimation, and DEGs analysis on the raw gene read counts. DEGs were identified based on thresholds of |log_2_FC| ≥ 1 and false discovery rate (FDR) < 0.05. Gene function was annotated using the GO, KEGG, KOG (Eukaryotic Orthologous Groups), NR (Non-redundant protein sequences), Swiss-Prot (Swiss-Prot protein sequence), Pfam (Protein family), and TrEMBL (Translated EMBL) databases. The genes were filtered based on the IQR (Interquartile Range) method using the varFilter function from the genefilter package in R, with a filtering threshold set at 0.5. Weighted gene co-expression network analysis (WGCNA) was then performed using WGCNA 1.71.

### 4.5. Metabolomics Analysis

Leaf samples from the tea plants of the Si group and the CK group subjected to drought treatment for 10 and 20 d were used for wide-targeted metabolomics analysis. Tea plant metabolites were extracted as previously described by Cao et al. [77]. The sample extracts were analyzed using a UPLC-ESI-MS/MS system (UPLC, ExionLC™ AD, https://sciex.com.cn/ (accessed on 23 May 2024)) coupled with tandem mass spectrometry. The analytical conditions were as follows. UPLC: column, Agilent SB-C18 (1.8 µm, 2.1 mm × 100 mm); mobile phase, solvent A, pure water with 0.1% formic acid, and solvent B, acetonitrile with 0.1% formic acid. The gradient elution program started at 95% A and 5% B, transitioning linearly to 5% A and 95% B over 9 min, maintained for 1 min, then adjusted to 95% A and 5% B within 1.1 min and held for another 2.9 min. The flow rate was maintained at 0.35 mL/min, the column temperature was 40 °C, and the injection volume was 2 μL. The effluent was alternately connected to an ESI-triple quadrupole-linear ion trap (QTRAP)-MS with ESI source temperature at 500 °C, ion spray voltage (IS) at 5500 V (positive ion mode)/−4500 V (negative ion mode), and ion source gases I (GSI), II (GSII), and curtain gas (CUR) set to 50, 60, and 25 psi, respectively, along with high collision-activated dissociation (CAD). Triple quadrupole mass spectrometer (QQQ) scans were performed in multiple reaction monitoring (MRM) mode with collision gas (nitrogen) set to medium. Specific MRM ion pairs were monitored based on the metabolites eluted in each period, and the chromatographic peaks of each MRM ion pair were integrated and corrected for quantitative analysis. The qualitative analysis of metabolites was based on public and MetWare MWDB databases, using retention time, m/z value, and fragmentation patterns for identification. DAMs were identified by variable importance in projection (VIP) values from the orthogonal partial least squares-discriminant analysis (OPLS-DA) model combined with fold change values, selecting metabolites with FC ≥ 2 or ≤0.5 and VIP > 1.

### 4.6. Quantitative Real-Time PCR (qRT-PCR)

To evaluate the accuracy of RNA-seq, 16 DEGs were validated by qRT-PCR. *CsACTIN* was used as the internal reference [78], and gene-specific primers listed in Table S12 were designed using Primer Premier 6.0 software. Gene expression profiles were analyzed using an SYBR Green PCR Kit (Wuhan Servicebio Biotechnology Co., Ltd., Wuhan, China) on a CFX384 instrument (Bio-Rad Laboratories, Inc., Hercules, CA, USA), following the protocol described by Cao et al. [79]. The relative expression levels of the selected genes were subsequently calculated using the 2^−ΔΔCt^ method as described by Livak and Schmittgen [80].

### 4.7. Statistical Analysis

Significant differences were assessed using an independent samples *t*-test conducted with SPSS 29.0.2.0 software. Principal component analysis (PCA), sample correlation analysis, hierarchical clustering analysis, and OPLS-DA were all performed through online platforms (https://cloud.metware.cn (accessed on 20 August 2024)). Graphs were created with GraphPad Prism 9.5.0, Cytoscape 3.9.1, and online tools (https://www.chiplot.online (accessed on 15 October 2024)).

## 5. Conclusions

In this study, we colonized tea plants with *S. indica* and observed mitigation of drought-induced damage. Transcriptomic and metabolomic analyses revealed that *S. indica* induces hormone signal transduction and MAPK signal transduction, forming a multi-layered regulatory network that activates drought-responsive TFs. The TFs further activate a series of drought-responsive genes and flavonoid biosynthetic genes. Changes in gene expression promote flavonoid accumulation and dynamic changes in osmoprotectants, thereby enhancing the drought tolerance of tea plants. In conclusion, this study preliminarily explored how *S. indica* enhances drought tolerance in tea plants and provides theoretical guidance for utilizing *S. indica* to improve crop drought tolerance in agricultural practices.

## Data Availability

The RNA-seq raw datasets generated during the current study have been deposited in NCBI repository under BioProject code PRJNA1218922. The datasets supporting the conclusions of this article are included within the article and its additional files.

## Supplementary Materials

The following supporting information can be downloaded at: https://www.mdpi.com/article/10.3390/plants14070989/s1, Figure S1: qRT-PCR verification of sixteen genes; Figure S2: (A) Principal component analysis (PCA) of 12 samples. (B) Correlation analysis of 12 samples; Figure S3: (A) KEGG pathways enrichment analysis of TFs in Si-D10d vs. CK-D10d. (B) KEGG pathways enrichment analysis of TFs in Si-D20d vs. CK-D20d; Figure S4: Top 5 KEGG pathways enrichment analysis of DEGs in the blue module. (B) Top 5 KEGG pathways enrichment analysis of DEGs in the brown module; Figure S5: (A) Volcano plot of differentially accumulated metabolites (DAMs) in different comparison groups. (B) Number of DAMs in different comparison groups. Table S1: Summary information of RNA-Seq; Table S2: 50 DEGs with the highest log_2_FC values in both Si-D10d vs. CK-D10d and Si-D20d vs. CK-D20d; Table S3: Identification of TFs in Si-D10d vs. CK-D10d; Table S4: Identification of TFs in Si-D20d vs. CK-D20d; Table S5: Information of DEGs in the blue module; Table S6: Information of DEGs in the brown module; Table S7: KEGG pathway enrichment analysis of DEGs in the blue module; Table S8: KEGG pathway enrichment analysis of DEGs in the brown module; Table S9: Information of DAMs in Si-D10d vs. CK-D10d; Table S10: Information of DAMs in Si-D20d vs. CK-D20d; Table S11: Shared upregulated and downregulated DAMs between Si-D10d vs. CK-D10d and Si-D20d vs. CK-D20d; Table S12: Primers used in this study.

## References

[1] Zhang Y., Keenan T.F., Zhou S. (2021). Exacerbated drought impacts on global ecosystems due to structural overshoot. Nat. Ecol. Evol..

[2] Gupta A., Rico-Medina A., Caño-Delgado A.I. (2020). The physiology of plant responses to drought. Science.

[3] Cao Y., Yang W., Ma J., Cheng Z., Zhang X., Liu X., Wu X., Zhang J. (2024). An integrated framework for drought stress in plants. Int. J. Mol. Sci..

[4] Shomali A., Das S., Arif N., Sarraf M., Zahra N., Yadav V., Aliniaeifard S., Chauhan D.K., Hasanuzzaman M. (2022). Diverse physiological roles of flavonoids in plant environmental stress responses and tolerance. Plants.

[5] Ahmed U., Rao M.J., Qi C., Xie Q., Noushahi H.A., Yaseen M., Shi X., Zheng B. (2021). Expression profiling of flavonoid biosynthesis genes and secondary metabolites accumulation in *Populus* under drought stress. Molecules.

[6] Lu W., Du Q., Xiao L., Lv C., Quan M., Li P., Yao L., Song F., Zhang D. (2021). Multi-omics analysis provides insights into genetic architecture of flavonoid metabolites in *Populus*. Ind. Crops Prod..

[7] Li B., Fan R., Sun G., Sun T., Fan Y., Bai S., Guo S., Huang S., Liu J., Zhang H. (2021). Flavonoids improve drought tolerance of maize seedlings by regulating the homeostasis of reactive oxygen species. Plant Soil.

[8] Fang Y., Xiong L. (2015). General mechanisms of drought response and their application in drought resistance improvement in plants. Cell Mol. Life Sci..

[9] Chakraborty U., Chakraborty B., Dey P., Chakraborty A.P. (2015). Role of microorganisms in alleviation of abiotic stresses for sustainable agriculture. Abiotic Stresses in Crop Plants.

[10] Kumar A., Tripti, Maleva M., Bruno L.B., Rajkumar M. (2021). Synergistic effect of ACC deaminase producing *Pseudomonas* sp. TR15a and siderophore producing *Bacillus aerophilus* TR15c for enhanced growth and copper accumulation in *Helianthus annuus* L.. Chemosphere.

[11] Lim J.H., Kim S.D. (2013). Induction of drought stress resistance by multi-functional PGPR *Bacillus licheniformis* K11 in pepper. Plant Pathol. J..

[12] Miozzi L., Vaira A.M., Brilli F., Casarin V., Berti M., Ferrandino A., Nerva L., Accotto G.P., Lanfranco L. (2020). Arbuscular mycorrhizal symbiosis primes tolerance to cucumber mosaic virus in tomato. Viruses.

[13] Tyagi J., Chaudhary P., Mishra A., Khatwani M., Dey S., Varma A. (2022). Role of endophytes in abiotic stress tolerance: With special emphasis on *Serendipita indica*. Int. J. Environ. Res..

[14] Verma S., Varma A., Rexer K.-H., Hassel A., Kost G., Sarbhoy A., Bisen P., Bütehorn B., Franken P. (1998). *Piriformospora indica*, gen. et sp. nov., a new root-colonizing fungus. Mycologia.

[15] Deshmukh S., Hückelhoven R., Schäfer P., Imani J., Sharma M., Weiss M., Waller F., Kogel K.-H. (2006). The root endophytic fungus *Piriformospora indica* requires host cell death for proliferation during mutualistic symbiosis with barley. Proc. Natl. Acad. Sci. USA.

[16] Mensah R.A., Li D., Liu F., Tian N., Sun X., Hao X., Lai Z., Cheng C. (2020). Versatile *Piriformospora indica* and its potential applications in horticultural crops. Hortic. Plant J..

[17] Li D., Bodjrenou D.M., Zhang S., Wang B., Pan H., Yeh K.W., Lai Z., Cheng C. (2021). The endophytic fungus *Piriformospora indica* reprograms banana to cold resistance. Int. J. Mol. Sci..

[18] Hosseini F., Mosaddeghi M.R., Dexter A.R. (2017). Effect of the fungus *Piriformospora indica* on physiological characteristics and root morphology of wheat under combined drought and mechanical stresses. Plant Physiol. Biochem..

[19] Tsai H.J., Shao K.H., Chan M.T., Cheng C.P., Yeh K.W., Oelmuller R., Wang S.J. (2020). *Piriformospora indica* symbiosis improves water stress tolerance of rice through regulating stomata behavior and ROS scavenging systems. Plant Signal Behav..

[20] Xu L., Wang A., Wang J., Wei Q., Zhang W. (2017). *Piriformospora indica* confers drought tolerance on *Zea mays* L. through enhancement of antioxidant activity and expression of drought-related genes. Crop J..

[21] Abdelaziz M.E., Atia M.A.M., Abdelsattar M., Abdelaziz S.M., Ibrahim T.A.A., Abdeldaym E.A. (2021). Unravelling the role of *Piriformospora indica* in combating water deficiency by modulating physiological performance and chlorophyll metabolism-related genes in *Cucumis sativus*. Horticulturae.

[22] Azizi M., Fard E.M., Ghabooli M. (2021). *Piriformospora indica* affect drought tolerance by regulation of genes expression and some morphophysiological parameters in tomato (*Solanum lycopersicum* L.). Sci. Hortic..

[23] Li L., Feng Y., Qi F., Hao R. (2023). Research progress of *Piriformospora indica* in improving plant growth and stress resistance to plant. J. Fungi.

[24] Yaghoubian Y., Goltapeh E.M., Pirdashti H., Esfandiari E., Feiziasl V., Dolatabadi H.K., Varma A., Hassim M.H. (2014). Effect of *Glomus mosseae* and *Piriformospora indica* on growth and antioxidant defense responses of wheat plants under drought stress. Agric. Res..

[25] Sun C., Johnson J.M., Cai D., Sherameti I., Oelmuller R., Lou B. (2010). *Piriformospora indica* confers drought tolerance in *Chinese cabbage* leaves by stimulating antioxidant enzymes, the expression of drought-related genes and the plastid-localized CAS protein. J. Plant Physiol..

[26] Meng X.H., Li N., Zhu H.T., Wang D., Yang C.R., Zhang Y.J. (2019). Plant resources, chemical constituents, and bioactivities of tea plants from the genus *Camellia* section *Thea*. J. Agric. Food Chem..

[27] Wang S., Gu H., Chen S., Li Y., Shen J., Wang Y., Ding Z. (2023). Proteomics and phosphoproteomics reveal the different drought-responsive mechanisms of priming with (Z)-3-hexenyl acetate in two tea cultivars. J. Proteom..

[28] Yue C., Cao H., Zhang S., Shen G., Wu Z., Yuan L., Luo L., Zeng L. (2023). Multilayer omics landscape analyses reveal the regulatory responses of tea plants to drought stress. Int. J. Biol. Macromol..

[29] Gu H., Wang Y., Xie H., Qiu C., Zhang S., Xiao J., Li H., Chen L., Li X., Ding Z. (2020). Drought stress triggers proteomic changes involving lignin, flavonoids and fatty acids in tea plants. Sci. Rep..

[30] Liu C., Wang Y., Wu Q., Yang T., Kuča K. (2020). Arbuscular mycorrhizal fungi improve the antioxidant capacity of tea (*Camellia sinensis*) seedlings under drought stress. Not. Bot. Horti Agrobot..

[31] Wu X.L., Hao Y., Lu W., Liu C.Y., He J.D. (2024). Arbuscular mycorrhizal fungi enhance nitrogen assimilation and drought adaptability in tea plants by promoting amino acid accumulation. Front. Plant Sci..

[32] Espareh A., Ghabooli M., Ershadi A., Karimi R., Movahedi Z. (2024). Calcium and *Serendipita indica* synergism enhance drought stress tolerance in grapevine by regulating some ABA-dependent and ABA-independent genes and morphophysiological parameters. Agric. Res..

[33] Ghabooli M., Kaboosi E. (2022). Alleviation of the adverse effects of drought stress using a desert adapted endophytic fungus and glucose in tomato. Rhizosphere.

[34] Rong Z.Y., Lei A.Q., Wu Q.S., Srivastava A.K., Hashem A., Abd_Allah E.F., Kuča K., Yang T. (2023). *Serendipita indica* promotes P acquisition and growth in tea seedlings under P deficit conditions by increasing cytokinins and indoleacetic acid and phosphate transporter gene expression. Front. Plant Sci..

[35] Cernava T., Chen X., Krug L., Li H., Yang M., Berg G. (2019). The tea leaf microbiome shows specific responses to chemical pesticides and biocontrol applications. Sci. Total Environ..

[36] Rivero R.M., Mittler R., Blumwald E., Zandalinas S.I. (2022). Developing climate-resilient crops: Improving plant tolerance to stress combination. Plant J..

[37] Khalid M., Rahman S.U., Huang D. (2019). Molecular mechanism underlying *Piriformospora indica*-mediated plant improvement/protection for sustainable agriculture. Acta Biochim. Biophys. Sin..

[38] Petrov P., Petrova A., Dimitrov I., Tashev T., Olsovska K., Brestic M., Misheva S. (2018). Relationships between leaf morpho-anatomy, water status and cell membrane stability in leaves of wheat seedlings subjected to severe soil drought. J. Agro Crop Sci..

[39] Hasanuzzaman M., Bhuyan M., Zulfiqar F., Raza A., Mohsin S.M., Mahmud J.A., Fujita M., Fotopoulos V. (2020). Reactive oxygen species and antioxidant defense in plants under abiotic stress: Revisiting the crucial role of a universal defense regulator. Antioxidants.

[40] Kaboosi E., Rahimi A., Abdoli M., Ghabooli M. (2023). Comparison of *Serendipita indica* inoculums and a commercial biofertilizer effects on physiological characteristics and antioxidant capacity of maize under drought stress. J. Soil. Sci. Plant Nutr..

[41] Liu D., Guo H., Yan L.-P., Gao L., Zhai S., Xu Y. (2023). Physiological, photosynthetic and stomatal ultrastructural responses of *Quercus acutissima* seedlings to drought stress and rewatering. Forests.

[42] Tyagi J., Varma A., Pudake R.N. (2017). Evaluation of comparative effects of arbuscular mycorrhiza (*Rhizophagus intraradices*) and endophyte (*Piriformospora indica*) association with finger millet (*Eleusine coracana*) under drought stress. Eur. J. Soil Biol..

[43] Del Rio D., Stewart A.J., Pellegrini N. (2005). A review of recent studies on malondialdehyde as toxic molecule and biological marker of oxidative stress. Nutr. Metab. Cardiovasc. Dis..

[44] Shah A., Smith D.L. (2020). Flavonoids in agriculture: Chemistry and roles in, biotic and abiotic stress responses, and microbial associations. Agronomy.

[45] He C., Du W., Ma Z., Jiang W., Pang Y. (2024). Identification and analysis of flavonoid pathway genes in responsive to drought and salinity stress in *Medicago truncatula*. J. Plant Physiol..

[46] Xu C., Wei L., Huang S., Yang C., Wang Y., Yuan H., Xu Q., Zhang W., Wang M., Zeng X. (2021). Drought resistance in qingke involves a reprogramming of the phenylpropanoid pathway and UDP-glucosyltransferase regulation of abiotic stress tolerance targeting flavonoid biosynthesis. J. Agric. Food Chem..

[47] Tattini M., Galardi C., Pinelli P., Massai R., Remorini D., Agati G. (2004). Differential accumulation of flavonoids and hydroxycinnamates in leaves of *Ligustrum vulgare* under excess light and drought stress. New Phytol..

[48] Liao Z., Liu X., Zheng J., Zhao C., Wang D., Xu Y., Sun C. (2023). A multifunctional true caffeoyl coenzyme A O-methyltransferase enzyme participates in the biosynthesis of polymethoxylated flavones in citrus. Plant Physiol..

[49] Xiao J., Muzashvili T.S., Georgiev M.I. (2014). Advances in the biotechnological glycosylation of valuable flavonoids. Biotechnol. Adv..

[50] Wang J., Hu Y., Guo D., Gao T., Liu T., Jin J., Zhao M., Yu K., Tong W., Ge H. (2024). Evolution and functional divergence of glycosyltransferase genes shaped the quality and cold tolerance of tea plants. Plant Cell..

[51] Zhao M., Jin J., Wang J., Gao T., Luo Y., Jing T., Hu Y., Pan Y., Lu M., Schwab W. (2022). Eugenol functions as a signal mediating cold and drought tolerance via UGT71A59-mediated glucosylation in tea plants. Plant J..

[52] Watkins J.M., Chapman J.M., Muday G.K. (2017). Abscisic acid-induced reactive oxygen species are modulated by flavonols to control stomata aperture. Plant Physiol..

[53] Tang X., Zhao C., Wen G., Wang W., Wang C., Sun Y., Bai X. (2014). Physiological mechanism for anthocyanins to strengthen the drought tolerance of plants. Agric. Sci. Technol..

[54] Lv X., Xiao H., Jie H., Ma Y., Long Z., He P., Xing H., Jie Y. (2024). Response of lignin and flavonoid metabolic pathways in *Capsicum annuum* to drought and waterlogging stresses. Not. Bot. Horti Agrobot..

[55] Tu Y., Liu F., Guo D., Fan L., Zhu Z., Xue Y., Gao Y., Guo M. (2016). Molecular characterization of flavanone 3-hydroxylase gene and flavonoid accumulation in two chemotyped safflower lines in response to methyl jasmonate stimulation. BMC Plant Biol..

[56] Castellarin S.D., Matthews M.A., Di Gaspero G., Gambetta G.A. (2007). Water deficits accelerate ripening and induce changes in gene expression regulating flavonoid biosynthesis in grape berries. Planta.

[57] Liu M., Li X., Liu Y., Cao B. (2013). Regulation of flavanone 3-hydroxylase gene involved in the flavonoid biosynthesis pathway in response to UV-B radiation and drought stress in the desert plant, *Reaumuria soongorica*. Plant Physiol. Biochem..

[58] Liu W., Feng Y., Yu S., Fan Z., Li X., Li J., Yin H. (2021). The flavonoid biosynthesis network in plants. Int. J. Mol. Sci..

[59] Ma D., Sun D., Wang C., Li Y., Guo T. (2014). Expression of flavonoid biosynthesis genes and accumulation of flavonoid in wheat leaves in response to drought stress. Plant Physiol. Biochem..

[60] Sperdouli I., Moustakas M. (2012). Interaction of proline, sugars, and anthocyanins during photosynthetic acclimation of *Arabidopsis thaliana* to drought stress. J. Plant Physiol..

[61] Liu S.C., Jin J.Q., Ma J.Q., Yao M.Z., Ma C.L., Li C.F., Ding Z.T., Chen L. (2016). Transcriptomic analysis of tea plant responding to drought stress and recovery. PLoS ONE.

[62] Zhang Q., Cai M., Yu X., Wang L., Guo C., Ming R., Zhang J. (2017). Transcriptome dynamics of *Camellia sinensis* in response to continuous salinity and drought stress. Tree Genet. Genomes.

[63] Wang M., Zhuang J., Zou Z., Li Q., Xin H., Li X. (2017). Overexpression of a *Camellia sinensis* DREB transcription factor gene (*CsDREB*) increases salt and drought tolerance in transgenic *Arabidopsis thaliana*. J. Plant Biol..

[64] Zhao L., Gao L., Wang H., Chen X., Wang Y., Yang H., Wei C., Wan X., Xia T. (2013). The *R2R3-MYB*, *bHLH*, *WD40*, and related transcription factors in flavonoid biosynthesis. Funct. Integr. Genom..

[65] Wu J., Lv S., Zhao L., Gao T., Yu C., Hu J., Ma F. (2023). Advances in the study of the function and mechanism of the action of flavonoids in plants under environmental stresses. Planta.

[66] Cheng C., Liu F., Wang B., Qu P., Liu J., Zhang Y., Liu W., Tong Z., Deng G. (2022). Influences of *Serendipita indica* and *Dictyophorae echinovolvata* on the growth and fusarium wilt disease resistance of banana. Biology.

[67] Kumar V., Sahai V., Bisaria V.S. (2011). High-density spore production of *Piriformospora indica*, a plant growth-promoting endophyte, by optimization of nutritional and cultural parameters. Bioresour. Technol..

[68] Cheng C., Li D., Qi Q., Sun X., Anue M.R., David B.M., Zhang Y., Hao X., Zhang Z., Lai Z. (2020). The root endophytic fungus *Serendipita indica* improves resistance of Banana to *Fusarium oxysporum* f. sp. *cubense* tropical race 4. Eur. J. Plant Pathol..

[69] Rai M., Acharya D., Singh A., Varma A. (2001). Positive growth responses of the medicinal plants *Spilanthes calva* and *Withania somnifera* to inoculation by *Piriformospora indica* in a field trial. Mycorrhiza.

[70] Yue C., Cao H.L., Wang L., Zhou Y.H., Huang Y.T., Hao X.Y., Wang Y.C., Wang B., Yang Y.J., Wang X.C. (2015). Effects of cold acclimation on sugar metabolism and sugar-related gene expression in tea plant during the winter season. Plant Mol. Biol..

[71] Wang L., Cao H., Qian W., Yao L., Hao X., Li N., Yang Y., Wang X. (2017). Identification of a novel bZIP transcription factor in *Camellia sinensis* as a negative regulator of freezing tolerance in transgenic arabidopsis. Ann. Bot..

[72] Sun B., Li W., Ma Y., Yu T., Huang W., Ding J., Yu H., Jiang L., Zhang J., Lv S. (2023). *OsGLP3-7* positively regulates rice immune response by activating hydrogen peroxide, jasmonic acid, and phytoalexin metabolic pathways. Mol. Plant Pathol..

[73] Yang C., Liu J., Qin X., Liu Y., Sui M., Zhang Y., Hu Y., Mao Y., Shen X. (2023). Effect of nitric oxide on browning of stem tip explants of *Malus sieversii*. Horticulturae.

[74] Cui X., Tang M., Li L., Chang J., Yang X., Chang H., Zhou J., Liu M., Wang Y., Zhou Y. (2024). Expression patterns and molecular mechanisms regulating drought tolerance of soybean [*Glycine max* (L.) Merr.] conferred by transcription factor gene *GmNAC19*. Int. J. Mol. Sci..

[75] Smith P.K., Krohn R.I., Hermanson G.T., Mallia A.K., Gartner F.H., Provenzano M.D., Fujimoto E.K., Goeke N.M., Olson B.J., Klenk D.C. (1985). Measurement of protein using bicinchoninic acid. Anal. Biochem..

[76] Xia E., Tong W., Hou Y., An Y., Chen L., Wu Q., Liu Y., Yu J., Li F., Li R. (2020). The reference genome of tea plant and resequencing of 81 diverse accessions provide insights into its genome evolution and adaptation. Mol. Plant.

[77] Cao H., Yue C., Luo L., Wang H., Shao H., Wu F., He L., Lucini L., Zeng L. (2024). Muti-omics analysis reveals the anthocyanin biosynthesis and accumulation mechanism in the hawk tea tree (*Litsea coreana* var. lanuginose). Food Biosci..

[78] Yu S., Zhu M., Li P., Zuo H., Li J., Li Y., Peng A., Huang J., Fernie A.R., Liu Z. (2024). Dissection of the spatial dynamics of biosynthesis, transport, and turnover of major amino acids in tea plants (*Camellia sinensis*). Hortic. Res..

[79] Cao H., Wang F., Lin H., Ye Y., Zheng Y., Li J., Hao Z., Ye N., Yue C. (2020). Transcriptome and metabolite analyses provide insights into zigzag-shaped stem formation in tea plants (*Camellia sinensis*). BMC Plant Biol..

[80] Livak K.J., Schmittgen T.D. (2001). Analysis of relative gene expression data using real-time quantitative PCR and the 2(-Delta Delta C(T)) Method. Methods.

