# Supplementary material for: Integrated Physiological, Transcriptomic, and Metabolomic Analysis Reveals Mechanism Underlying the Serendipita indica-Enhanced Drought Tolerance in Tea Plants
Source: Plants (Basel). 2025 Mar 21;14(7):989. doi: 10.3390/plants14070989 (PMC11990811; doi:10.3390/plants14070989)
Supplement: Supplementary file 1 [file plants-14-00989-s001.zip › plants-3496527-supplementary.pdf]

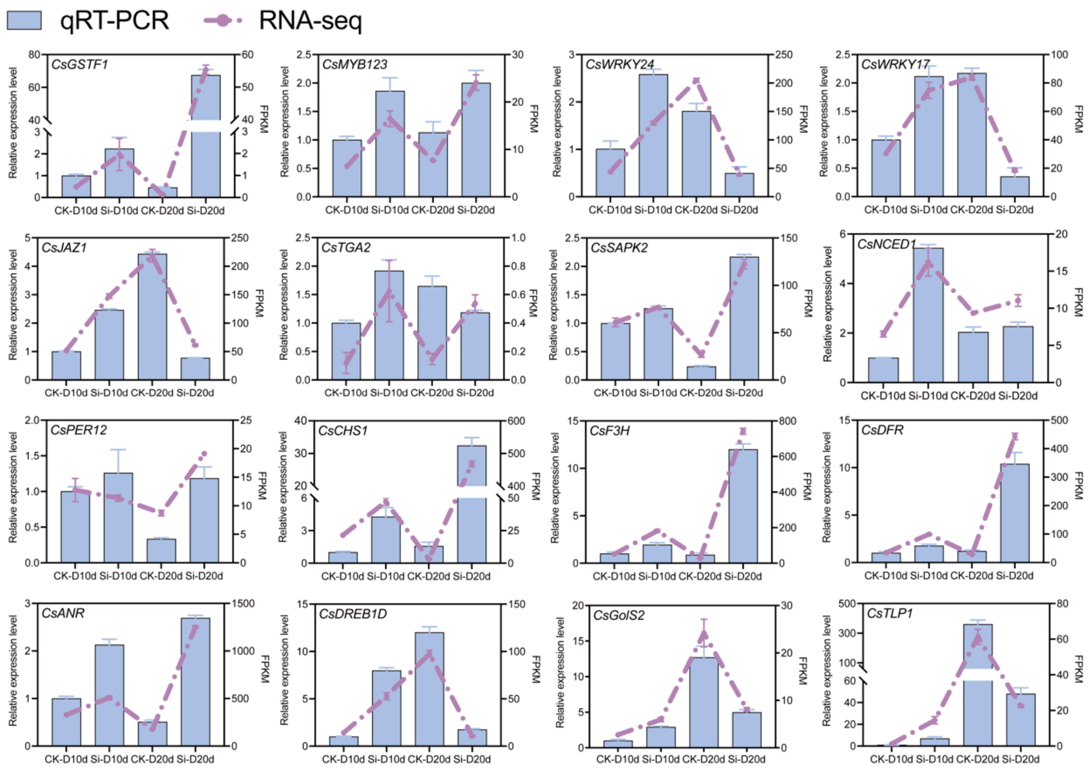

Figure S1. qRT-PCR verification of sixteen genes.

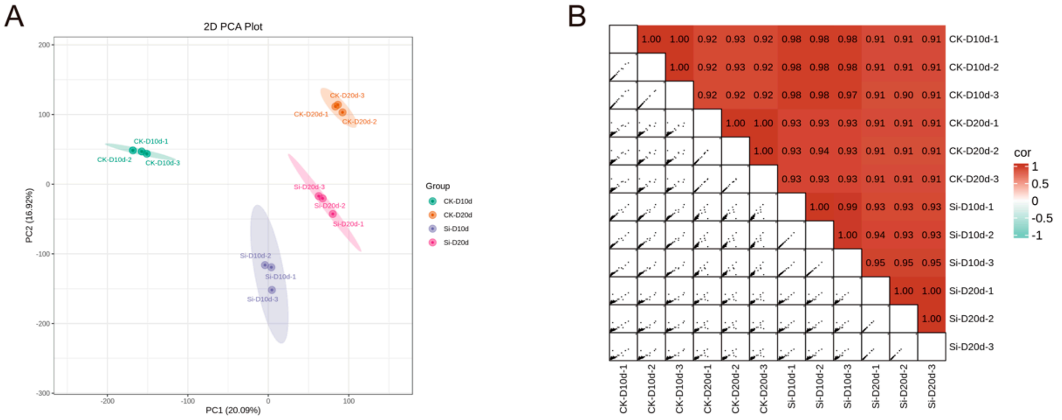

Figure S2. (A) Principal component analysis (PCA) of 12 samples. (B) Correlation analysis of 12 samples.

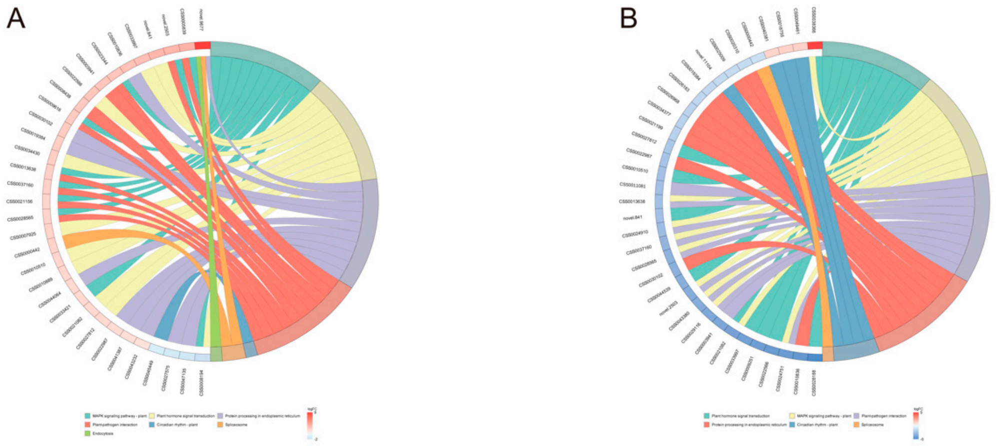

Figure S3. (A) KEGG pathways enrichment analysis of TFs in Si-D10d vs. CK-D10d. (B) KEGG pathways enrichment analysis of TFs in Si-D20d vs. CK-D20d.

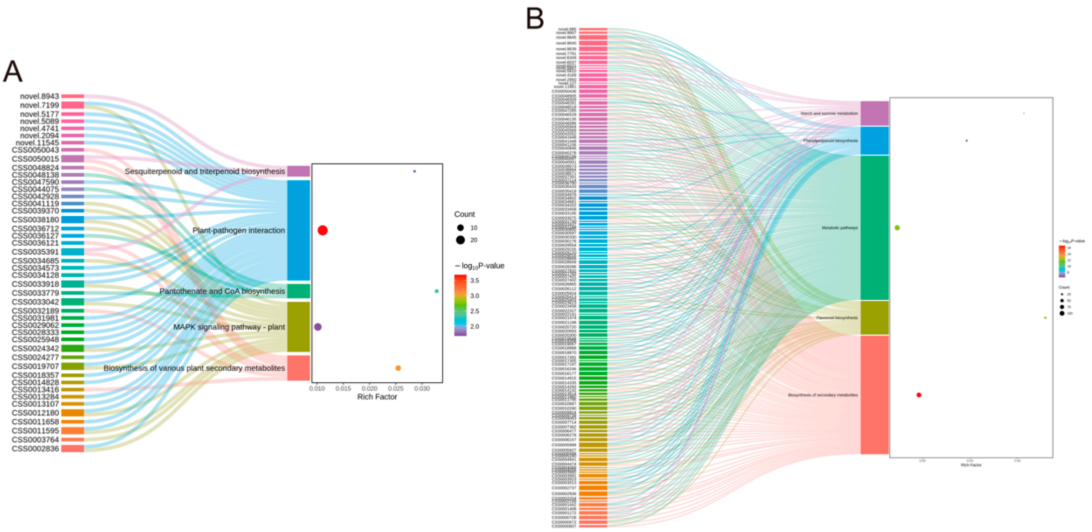

Figure S4. (A) Top 5 KEGG pathways enrichment analysis of DEGs in the blue module. (B) Top 5 KEGG pathways enrichment analysis of DEGs in the brown module.

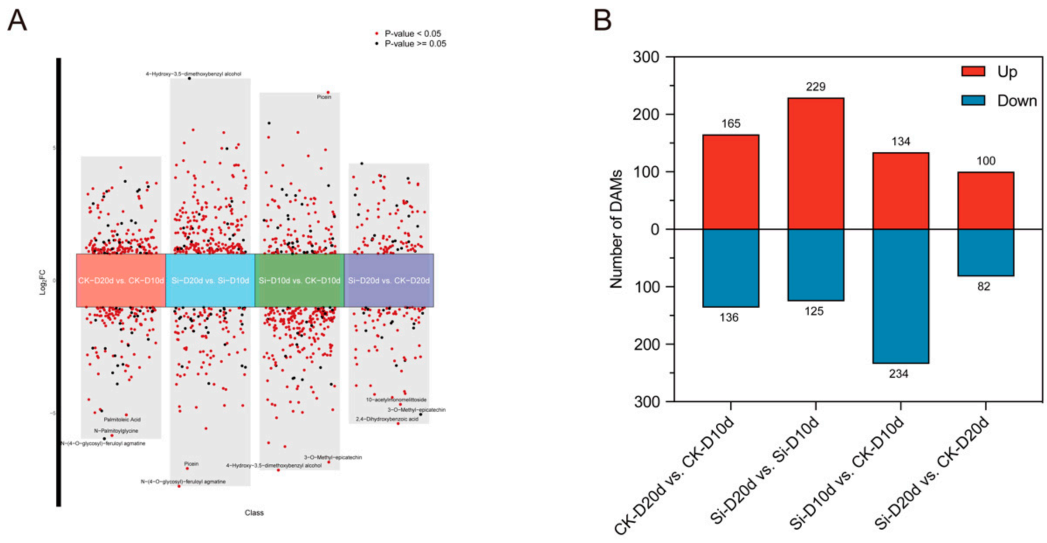

Figure S5. (A) Volcano plot of differentially accumulated metabolites (DAMs) in different comparison groups. (B) Number of DAMs in different comparison groups.
